# Detection of the Endangered Siamese Bat Catfish (*Oreoglanis siamensis* Smith, 1933) in Doi Inthanon National Park Using Environmental DNA

**DOI:** 10.3390/ani13030538

**Published:** 2023-02-03

**Authors:** Thanatrinan Rodpai, Chatmongkon Suwannapoom, Maslin Osathanunkul

**Affiliations:** 1 Department of Biology, Faculty of Science, Chiang Mai University, Chiang Mai 50200, Thailand; 2 School of Agriculture and Natural Resources, University of Phayao, Phayao 56000, Thailand; 3 Research Center in Bioresources for Agriculture, Industry and Medicine, Chiang Mai University, Chiang Mai 50200, Thailand

**Keywords:** endemic and endangered fish, nondestructive methods, environmental DNA, conservation plan, Thai national park

## Abstract

**Simple Summary:**

Using traditional methods for surveying and monitoring Siamese bat catfish (*Oreoglanis siamensis* Smith, 1933), an endangered and endemic species in Thailand, is difficult. In this study, the eDNA-based method was used to confirm the *O. siamensis* habitat and its presence in the Doi Inthanon National Park, Chiang Mai, Thailand. Water samples were collected, and eDNA was analyzed by real-time PCR with species-specific primers. The eDNA of *O. siamensis* was detected in 12 samples out of 15 samples, inferring its distribution in the rivers of the Doi Inthanon National Park. The results showed that the eDNA-based approach can successfully detect *O. siamensis* in 300 mL turbid water samples. This information may be beneficial for the species management plan. The success of the eDNA-based method in *O. siamensis* detection indicates the usefulness of this method for rare species surveys in unfavorable environments.

**Abstract:**

Siamese bat catfish (*Oreoglanis siamensis* Smith, 1993) has been listed as an endangered species, and its abundance has been severely declining due to habitat degradation and overfishing. To establish an appropriate management strategy, it is crucial to gain information about the distribution of this endangered species. As *O. siamensis* live under rocks in streams, detecting their presence is difficult. Recently, environmental DNA (eDNA)–based detection has been demonstrated to be a valid tool for monitoring rare species, such as *O. siamensis*. Therefore, this study developed an eDNA assay targeting a 160 bp fragment of the *COI* region to detect the presence of this species in its natural habitat. An amount of 300 mL of water samples (0.7 μm filtered) were collected from 15 sites in the Mae Klang sub-basin, where this fish species was visually detected at two locations. *O. siamensis* eDNA was detected at 12 of the 15 sites sampled with varying concentrations (0.71–20.27 copies/mL), including at the sites where this species was visually detected previously. The developed *O. siamensis* eDNA assay was shown to be effective for detecting the presence of this endangered species in the Klang Phat and Klang Rivers within the Doi Inthanon National Park.

## 1. Introduction

Siamese bat catfish (*Oreoglanis siamensis* Smith, 1993) is an endemic species that inhabits in streams in the Doi Inthanon National Park, Chiang Mai Province, Thailand. It has been listed as an endangered species (EN) on the IUCN Red List since 2013 [1]. *Oreoglanis siamensis* is one ecologically important species and can be used as an indicator of the integrity of the ecosystem as it lives in clear and swift-running water with high oxygen content, and it is very sensitive to chemicals [2,3,4]. Although it is registered as a protected species under the National Park Act in Thailand, over the past few years, *O. siamensis* abundance has been severely declining, with causes ranging from habitat loss to overfishing [5]. Therefore, an effective conservation plan is urgently required to preserve this species, once the risk of disappearance from these areas is high.

The study of population density and dispersion is an integral part of endangered species conservation [6,7,8]. It is essential to have information regarding species’ abundance and distribution to develop an appropriate management plan. Conventional fish survey approaches using capture-based sampling, such as electrofishing, seining, trapping, and netting, have traditionally provided accurate information for fisheries management and conservation [9]. However, these survey methods are time and labor-intensive and need the participation of several fisheries professionals, having limited sensitivity and detection biases [10,11,12]. In addition, conventional techniques can be destructive, interfering with the species and the environment. As a result, developing well-informed management decisions provided by these species’ distribution surveys is sometimes difficult [13,14]. As *O. siamensis* numbers are still in decline and there is difficulty in detecting specimens using conventional methods, a more powerful or sensitive detection approach could give a better chance to monitor this species and develop appropriate conservation plans.

Previous studies have demonstrated that fish environmental DNA (eDNA) can be detected in ponds [15,16], streams [17,18], rivers [19,20], and seas [21,22]. Recently, there has been growing circumstantial evidence that surveillance of endangered or difficult-to-detect species using eDNA-based detection can be advantageous (e.g., [23,24,25,26,27,28]). Environmental-DNA-based methods have been shown to be more sensitive and effective than traditional sampling techniques for detecting rare or elusive species [29,30]. Using eDNA-based detection approaches can overcome the limitations of traditional methods, particularly the detection bias, which can result in the absence of detection of target animals even if they are present [31]. Environmental DNA analysis also requires less sampling effort and can cost less than triple-pass electrofishing [12]. In addition, eDNA-based detection helps to minimize associated damage to endangered species and the environment [31,32].

Thus, in this study, an eDNA-based method was developed for monitoring *O. siamensis* in the Doi Inthanon National Park, Chiang Mai Province, Thailand, and the results were compared with historical visual detections of this species in the study area. This technique could be a reliable and cost-effective surveillance tool that can provide more robust and large-scale data to enable effective conservation and management devoted to *O. siamensis*.

## 2. Materials and Methods

### 2.1. Primers and Probe Development

**Figure 1:**
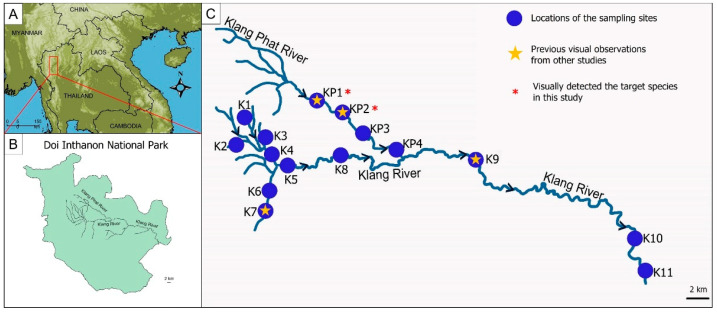
Locations of the sampling sites with indication of previous visual observation. (**A**) Map of Thailand, (**B**) a general map of the Doi Inthanon National Park with details of the Klang Phat River and Klang River, (**C**) location of the sampling sites (N = 15) and visual detection of the target species in this study (N = 2) across the Klang Phat River and Klang River in the Doi Inthanon National Park, Chiang Mai, Thailand.

Reference sequences from two mitochondrial loci, cytochrome oxidase subunit 1 gene (*COI*) and cytochrome b (*Cytb*), and two nuclear loci, *12S rRNA* (*12S*) and *16S rRNA* (*16S*), of related species were downloaded from GenBank (https://www.ncbi.nlm.nih.gov/genbank/ (accessed on 14 February 2020)), while sequences of the targeted species were generated as part of this study (Supplementary Table S1). Tissue samples of the three *O. siamensis* specimens were provided by the Department of Fisheries, Thailand (collected near the K7 site, Figure 1), and mucus samples collected from three specimens found at the KP1 site (Figure 1) were used for DNA extraction. Total DNA was extracted from the samples using the DNeasy Blood and Tissue Kit (Qiagen, Hilden, Germany), according to the manufacturer’s protocol. *COI* and *Cytb* were then amplified and sequenced using LCO1490 and HCO2198 primers [33] and L1472 and H1514 primers [34], respectively. *12S* and *16S* were amplified and sequenced using 12Sa and 12Sb primers [35] and 16Sar and 16Sbr primers [35], respectively. All generated sequences were deposited in GenBank with the following accession numbers: MZ753673-78 for the *COI* region, MZ773400-05 for the *Cytb* region, and MZ766143 and MZ766142 for the *12S* and *16S* regions.

All sequences were aligned in MEGA X [36]. Primers were designed from the *COI* sequence as the *Cytb*, *12S*, and *16S* sequences did not have enough mismatches with the related species. Species-specific primers and a minor-groove binding (MGB) probe incorporating a 5′ FAM reporter dye and a 3′ nonfluorescent quencher were designed to amplify a 160 bp fragment (Table 1). Cross amplification of unrelated species was tested using Primer Blast (https://www.ncbi.nlm.nih.gov/tools/primer-blast/ (accessed on 20 February 2020)) and BLASTn (Basic Local Alignment Search Tool) (Table 2). The designed primers and probe were also tested for amplification of targeted species and for cross amplification with closely related (*O. colurus* and *O. vicinus*) and nontarget species, including *Barbonymus gonionotus*, *Channa aurolineatus*, *C. micropeltes*, *C. striata*, *Chitala ornata*, *Ceratogarra cambodgiensis*, *Hypsibarbus malcolmi*, *Labiobarbus spilopleura*, *Pangasianodon gigas*, *P. hypophthalmus*, *Pangasius bocourti*, *P. larnaudii*, *Probarbus jullieni*, and *Puntioplites proctozysron*.

### 2.2. Study Sites

*Oreoglanis siamensis* are currently located in the Klang Phat River and Klang River in the Doi Inthanon National Park, Chiang Mai, Thailand (Figure 1), according to physical captures by local people and visual detection. The species live in shaded areas of highland streams, on the bottom of cool, clear, and fast-flowing streams with pebbles and stones. Based on the behavior and habitat preference of the target species and its visual detection and local accessibility, 15 sampling sites were selected for water samplings (Figure 1 and Supplementary Table S2). Surveys were performed once a year (2019–2021). Samples were collected in and beyond the currently known distribution boundaries of *O. siamensis* in the Doi Inthanon National Park. This research was conducted under the permission of the Department of National Parks, Wildlife, and Plant Conservation, Ministry of Natural Resources and Environment, Thailand, and the Department of Fisheries, Ministry of Agriculture and Cooperatives, Thailand.

### 2.3. Water Sampling and eDNA Extraction

Three surface water samples (300 mL) from each site were collected in previously decontaminated buckets (10% bleach rinse, followed by two distilled water rinses) while wearing gloves that were changed between sites to prevent cross contamination.

Water samples were immediately filtered in the field using a 50 mL sterile BD Luer-Lok™ Syringe (BD, Franklin Lakes, NJ, USA) with a 0.7 μm filter (Whatman International Ltd., Maidstone, UK). Negative controls were assessed on each sampling day by filtering 300 mL of deionized water. Each filter was then put in a 1.5 mL microcentrifuge tube and placed in a storage box containing dry ice. The storage box was transported to the laboratory and stored at −20 °C.

Environmental DNA contained in each filter was extracted using the Qiagen DNeasy Blood and Tissue Kit (Qiagen, Hilden, Germany) with a slight modification to the protocol from the manufacturer’s protocol following Osathanunkul and Minamoto (2020 and 2021) [37,38]. To remove any inhibitors, all DNA samples were cleaned up using the OneStep PCR Inhibitor Removal Kit (Zymo Research, Irvine, CA, USA) [37,38,39] following the manufacturer’s protocol and stored at −20 °C until further analyses.

### 2.4. qPCR Assay

The qPCR assay was performed following Osathanunkul and Minamoto (2020 and 2021) [37,38]. Briefly, each eDNA qPCR amplification was carried out in five replicates per site per sampling year at a final volume of 20 μL using 2.0 μL of DNA template, 10.0 μL of 2× TaqMan Environmental Master Mix 2.0 (Thermo Fisher Scientific, Waltham, MA, USA), 900 nM each of the F/R primers, and 125 nM of the probe. Samples were run using Rotor-Gene Q (Qiagen, Hilden, Germany) under the following conditions: a 10 min initial incubation at 95 °C, followed by 55 cycles of denaturation at 95 °C for 15 s and annealing/extension at 60 °C for 1 min. Positive (gDNA) and no template (Milli-Q water) controls were included on each qPCR assay plate. MIQE guidelines were consulted to ensure that all information relevant to presence/absence eDNA assays was reported. Positive detections were confirmed by sequencing of the amplicons (Celemics, Inc., Seoul, Korea). The copies/mL values for *O. siamensis* eDNA concentrations were reported. A standard dilution series of synthesized target gene fragments (Integrated DNA Technologies Pte. Ltd., Singapore) with known copy numbers was used to generate a standard curve and measure detection and quantification limits [26,28]. With 12 technical replicates used for each dilution step, the standard concentrations were adjusted to 12,500, 1250, 125, 12.50, 1.25, and 0.125 copies/mL. The efficiency for the qPCR assay was 99% (y = −3.318x + 42.784; R^2^ = 0.99). This regression equation was used to convert the quantification cycle data from the qPCR product (i.e., the PCR cycle at which the target is considered positively amplified in a given sample) into the DNA concentration in a particular sample, for example, [40,41]. Both DNA extract from the mucus of *O. siamensis* and the synthesized fragment were used as positive controls.

### 2.5. Fish Population

Sequences of four DNA regions, including *COI* (651 bp), *Cytb* (432 bp), *12S* (331 bp), and *16S* (525 bp), were generated from DNA extracted from tissue and mucus samples of *O. siamensis* collected from the Klang Phat River (KP1) and Klang River (K7). The sequences were aligned using MEGA X to compare the differences in fish population from those two rivers.

## 3. Results

### 3.1. Specificity of Designed Primers and Probe

Partial *COI* region sequences of *O. siamensis* were generated and deposited in GenBank. The specific primers and probe were designed based on sequences generated in this study and retrieved from GenBank. A 160 bp DNA fragment specific to *O. siamensis* with the designed primer pair was successfully amplified. To determine primer specificity, primer pairs were tested in silico (MEGA X) and in vitro (PCR and qPCR) [42,43]. The designed primers were found to be specific to *O. siamensis,* as no amplification was found with other tested species.

### 3.2. eDNA Detection at Sampling Sites

Positive eDNA detections for *O. siamensis* were observed at 12 sites with varying concentrations (Table 3). The positive detections were confirmed by sequencing of the amplicons (Supplementary Table S3). The highest average eDNA concentration was 20.27 copies/mL at the KP1 site, while the lowest was found at K4 (0.71 copies/mL). No *O. siamensis* eDNA was detected in three sites (K3, K6, and K11). No *O. siamensis* eDNA was detected in any of the negative control samples. In this study, the eDNA was detected at all sites where the target species was visually observed during this and a previous study [4].

### 3.3. qPCR Assay Sensitivity

The detection limit (LD) with a 95% confidence interval and the quantification limit (LQ) with a threshold of 35% were used to represent the sensitivity of the qPCR analysis. The qPCR assay of *O. siamensis* had an LD of 0.48 copies/mL and an LQ of 0.48 copies/mL.

### 3.4. Fish Population

As the eDNA of *O. siamensis* was detected in both the Klang Phat River and Klang River, we wanted to confirm whether the fish inhabiting both rivers belong to the same population. Performed analyses indicated no differences between samples collected from the Klang Phat and Klang Rivers for the *COI*, *Cytb*, and *16S* regions and only 1 bp difference in the *12S* region (Supplementary Figure S1).

## 4. Discussion

In the current study, a primer–probe set targeting an amplified product size of 160 bp for eDNA detection of *O. siamensis* was successfully developed, and the specificity of the primers for this species was confirmed both by in silico and in vitro analyses [44,45]. Although some studies suggest that short amplified fragments (≤150 bp) result in more successful amplification of degraded DNA, for example, [19,46,47,48,49], others found that the target amplicon size has no effect on eDNA detection, for example, [50,51,52]. Nonetheless, the amplicon size may affect the difference in eDNA detection between lotic and lentic systems, whose shorter target product might be better for samples collected from rivers or streams [51].

In this study, the *COI* assay can successfully amplify the *COI* region of the *O. siamensis* eDNA. Moreover, it is one of the commonly used markers for targeted species detection from eDNA, which shows high levels of conservation within species and decreased levels of genetic variation among different species. It is also typically used as a highly effective species barcode for fish identification [53]. In other studies, the *COI* region was successfully used for species detection, such as *Galaxiella pusilla* [16], *Anoxypristis cuspidata* [51], and *Salmo salar* [52]. However, the marker selection relied on the availability of sequences representing barcode regions [54].

The eDNA analysis in this study was in agreement with previously reported visual observations in that eDNA detection was positive at the sites where *O. siamensis* was previously reported (KP1, KP2, K7, and K9). A previous study by Ng and Rainboth (2001) reported that *O. siamensis* was also detected in Wachirathan Waterfall, Doi Inthanon National Park, Chiang Mai, Thailand, [4] at the K9 sampling site (Figure 1). A higher concentration of the *O. siamensis* eDNA was found at three sites, including KP1, KP2, and KP4. On the other hand, the eDNA concentration was lower at the other sites due to the lower species abundance, which might lead to the occurrence of false negatives [55,56,57]. Several factors play a role in eDNA detection error (false negative), such as insufficient assay sensitivity to detect a target eDNA, numerous freeze-thawing, and long-term storage of samples [12,58]. Some researchers suggest that false negatives could be overcome by increasing the filtering water volume or using multiple filters [59,60]. A previous study reported that eDNA detection rates in streams were increased by filtering 1–2 L of water [61,62]. Filtering a larger amount of water using a larger pore size may be the simplest technique to boost detection in systems with poor overall detection rates [63,64,65]. However, in several studies that collected turbid water samples, volumes of less than 300 mL were successfully used, for example, [12,51,66,67]. In this study, a 300 mL filtered water volume is potentially too low to have captured very low abundant eDNA of the target species in 3/15 sites (K3, K6, and K11). Therefore, the consistency of nondetection at these 3/15 sites across all 3 sampling years (2019–2021) suggests that target species abundance is either very low or actually absent from these sites (i.e., unable to conclusively determine whether these are actually false negatives due to low abundance or true negatives).

**Figure 2:**
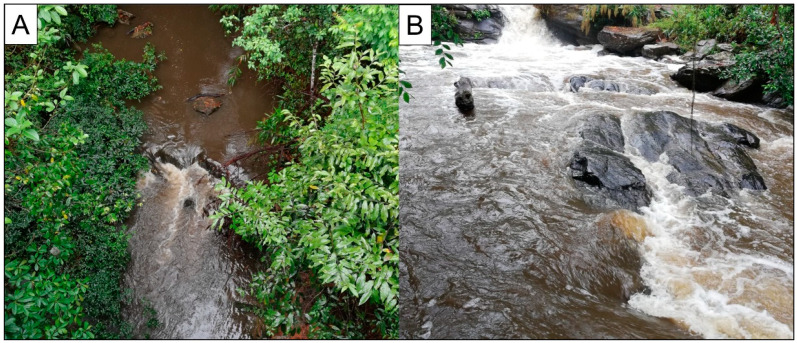
Turbid water at sampling sites. (**A**) KP4 from the Klang Phat River and (**B**) K11 from the Klang River.

For turbid water samples, filtering a large volume of water using filters with a small pore size (e.g., <1 μM) is very difficult because of clogging (Figure 2). Thus, in this study, 300 mL was the maximum volume used for sample filtration due to the water turbidity. Another factor that should be considered when dealing with eDNA detection is eDNA transportation in lotic ecosystems, as it might have an effect not only on estimating the presence of species but also on the geographical dispersion of aquatic animals. Stream flow decreases DNA concentration by dilution and dispersion effects, making it more difficult to detect the eDNA especially of low abundance species. Nukazawa et al. (2018) suggested that in case of low abundance target species, the survey should predict the detectable distance and study the characteristics of the target sampling source [68]. To increase the concentration of the eDNA, riverbed sediment should be collected as described by Turner et al. (2015), suggesting that its eDNA concentration is higher when compared with surface water [65]. Moreover, DNA concentrations in streams and rivers are directly influenced by the equilibrium between DNA entering and leaving the system as well as downstream flow [69,70]. However, we suggest that future studies should be concerned about other factors that can affect the eDNA concentration, including transportation [71], DNA degradation [72], dynamics in rivers [73], and abundance of target species [18], to improve the sampling design. Variations in river size, flow, and structure also affect the eDNA transportation; therefore, estimating average transport distances at each sampling location could help improve sample quality [70].

An eDNA-based tool for *O. siamensis* detection was successfully developed in this study. To minimize further loss of the species, the effects of anthropogenic disturbance on its population is another question that needs to be addressed [74]. Environmental DNA has the ability to fill in the blanks and might give answers to this issue. Notably, *O. siamensis* have been declining markedly, with reasons ranging from habitat degradation to locals catching the fish for food. Thus, they are protected by law along with 25 other fish species in Thailand. Their habitats are in areas where the National Park Act has been in action, so that hunting animals or catching fish is strictly prohibited. A lack of knowledge or awareness that some species that inhabit the rivers within the Doi Inthanon National Park (e.g., fish) are rare and endemic and, thus, need to be conserved could lead to failure to protect them. Improving knowledge and local awareness of the decline of endangered fish populations can benefit the conservation management in key areas (e.g., national parks). The eDNA assay developed in this study can be utilized by government agencies and local communities to develop an effective species management plan and raise awareness about *O. siamensis* conservation.

## 5. Conclusions

Here, a *COI*-targeting eDNA-based assay was successfully used to confirm the presence of the endangered *O. siamensis* in both the Klang Phat and the Klang River of the Doi Inthanon National Park. This study demonstrates that eDNA surveys for *O. siamensis* provide an easy, fast, and sensitive way to monitor this endangered species.

## Figures and Tables

**Table 1:** Species-specific primers and probe designed to amplify a 160 bp fragment of the *COI* region of *O. siamensis*.

Primer Name	Type	Length (bp)	Primer Sequence 5′–3′	Amplicon Size
Osi 434 COI-F	Forward	23	CCTTGCAGGTGTATCGTCTATTC	160 bp
Osi 576 COI-R	Reverse	19	AGCTGCCAAGACTGGTAGT	
Osi COI-PR	Probe	27	CCTCCAGCAATTTCCCAATACCAAACC	

**Table 2:** Homology of the query to the primers and probe and percentage identity as a function of the number of matching base sites divided by 69 (total number of base sites across the primer pair and probe). The base site homology between the query and the primer is shown as a dot.

Species	Forward	Reverse	Probe	Identity (%)	Accession Number
*Oreoglanis siamensis*	·······················	···················	···························	100	MZ753672
*Oreoglanis insignis*	·········G············T	···G······C·A··T··C	··C········C··A········G···	84	DQ508082
*Oreoglanis* sp.	·········A·····A··C····	···············T··C	··C········C···············	90	DQ846711
*Oreoglanis immaculatus*	······G··G··G·········T	···G··C···C····T··C	··C··G·····C··A··G········T	78	JQ859840
*Pareuchiloglanis anteanalis*	T········A·····A·······	·T········C·A··T··C	··C··T·····C··G············	82	DQ508085
*Pareuchiloglanis sinensis*	T········A·····A·······	·T········C·A··T··C	··C··T·····C··G············	82	MF122630
*Pseudexostoma yunnanense*	T········G·C···A·······	······G···C·A··T··C	··C········C··A········G··T	79	KU987335
*Pseudexostoma longipterus*	T········G·C···A·······	G·········C·A··T··C	···········C··A···········T	82	KU987301

**Table 3:** eDNA detection (2019–2021), average *O. siamensis* eDNA concentration (copies/mL), and visual observation record at each sampling site.

Site	River	eDNA Detection			eDNA Detection (Average Concentration, Copies/mL)	Visual Detection in This Study	Visual Detection from Other Studies	References
		2019	2020	2021				
KP1	Klang Phat	√	√	√	20.27	√	√	Local reports
KP2		√	√	√	17.58	√	√	Local reports
KP3		√	√	√	2.60	x	x	-
KP4		√	√	√	12.00	x	x	-
K1	Klang	√	√	√	1.00	x	x	-
K2		√	√	√	1.03	x	x	-
K3		x	x	x	-	x	x	-
K4		√	√	√	0.71	x	x	-
K5		√	√	√	1.86	x	x	-
K6		x	x	x	-	x	x	-
K7		√	√	√	1.23	x	√	Local reports
K8		√	√	√	1.71	x	x	-
K9		√	√	√	1.73	x	√	[4]
K10		√	√	√	3.94	x	x	-
K11		x	x	x	-	x	x	-

## Data Availability

The datasets generated and/or analyzed during the current study are available in the GenBank repository (accession numbers were provided in Supplementary Tables S1 and S3).

## Supplementary Materials

The following supporting information can be downloaded at: https://www.mdpi.com/article/10.3390/ani13030538/s1, Figure S1: Sequence alignments of *O. siamensis* from the Klang and Klang Phat rivers; Table S1: GenBank accession numbers of species were used for primer design in this study; Table S2: Geographic coordinates of the sampling sites; Table S3: Overview of sequence alignments (160 bp) between positive qPCR amplicons from eDNA assays and *O. siamensis* gDNA sequence. One eDNA amplicon was randomly selected for sequencing from each sampling site for each sampling year (gray highlighted).
