# Supplementary material for: Detection of the Endangered Siamese Bat Catfish (Oreoglanis siamensis Smith, 1933) in Doi Inthanon National Park Using Environmental DNA
Source: Animals (Basel). 2023 Feb 3;13(3):538. doi: 10.3390/ani13030538 (PMC9913137; doi:10.3390/ani13030538)
Supplement: Supplementary file 1 [file animals-13-00538-s001.zip › animals-1939836-Supplementary Figure S1.pdf]

Supplementary Figure S1. Sequence alignments of *O. siamensis* from Klang and Klang Phat rivers. A. COI region, B. *Cytb* region, C. 12S rRNA region, and D. 16S rRNA region.

A.

Name	GAGCCTGAGCTGGAATGGTCGGCACCGCCCTCAG
1. Oreoglanis siamensis from Klang river	....
2. Oreoglanis siamensis from Klang Phat river	....
Name	CCTCTTAATCCGAGCGGAGCTGGCCCAGCCAGGA
1. Oreoglanis siamensis from Klang river	....
2. Oreoglanis siamensis from Klang Phat river	....
Name	ACTCTTTTGGGTGACGATCAAATTTATAATGTTA
1. Oreoglanis siamensis from Klang river	....
2. Oreoglanis siamensis from Klang Phat river	....
Name	TCGTCACTGCACATGCCTTCGTTATAATCTTCTT
1. Oreoglanis siamensis from Klang river	....
2. Oreoglanis siamensis from Klang Phat river	....
Name	TATAGTAATACCTGTAATAATTGGAGGCTTCGGC
1. Oreoglanis siamensis from Klang river	....
2. Oreoglanis siamensis from Klang Phat river	....
Name	AACTGACTCGTCCCTCTAATAATTGGAGCCCCAG
1. Oreoglanis siamensis from Klang river	....
2. Oreoglanis siamensis from Klang Phat river	....
Name	ACATAGCATTTCCTCGAATAAATAACATAAGCTT
1. Oreoglanis siamensis from Klang river	....
2. Oreoglanis siamensis from Klang Phat river	....
Name	CTGACTTCTGCCCCCATCCTTTCTCCTACTCCTG
1. Oreoglanis siamensis from Klang river	....
2. Oreoglanis siamensis from Klang Phat river	....
Name	GCCTCTTCTGGAATTGAAGCAGGCGCAGGAACAG
1. Oreoglanis siamensis from Klang river	....
2. Oreoglanis siamensis from Klang Phat river	....
Name	GCTGAACTGTCTACCCTCCCTTAGCAGGAAACTT
1. Oreoglanis siamensis from Klang river	....
2. Oreoglanis siamensis from Klang Phat river	....
Name	AGCACACGCAGGGCCTTCTGTAGACCTAACCATT
1. Oreoglanis siamensis from Klang river	....
2. Oreoglanis siamensis from Klang Phat river	....
Name	TTCTCATTACACCTTGCAGGTGTATCGTCTATTC
1. Oreoglanis siamensis from Klang river	....
2. Oreoglanis siamensis from Klang Phat river	....
Name	TAGGAGCCATTAATTTTATCACAACAATTATTAA
1. Oreoglanis siamensis from Klang river	....
2. Oreoglanis siamensis from Klang Phat river	....
Name	TATAAAACCTCCAGCAATTTCCCAATACCAAACC
1. Oreoglanis siamensis from Klang river	....
2. Oreoglanis siamensis from Klang Phat river	....
Name	CCCTTATTCGTATGAGCTGTCTTAATTACAGCTG
1. Oreoglanis siamensis from Klang river	....
2. Oreoglanis siamensis from Klang Phat river	....
Name	TACTACTACTGTTATCACTACCAGTCTTGGCAGC
1. Oreoglanis siamensis from Klang river	....
2. Oreoglanis siamensis from Klang Phat river	....
Name	TGGCATCACAATACTTTTAACAGATCGAAACTTA
1. Oreoglanis siamensis from Klang river	....
2. Oreoglanis siamensis from Klang Phat river	....
Name	AATACCACCTTCTTTGATCCCTCAGGGGGAGGAG
1. Oreoglanis siamensis from Klang river	....
2. Oreoglanis siamensis from Klang Phat river	....
Name	ACCCAATCCTTTACCAACACCTATTTTGATTTTT
1. Oreoglanis siamensis from Klang river	....
2. Oreoglanis siamensis from Klang Phat river	....
Name	TGGTC
1. Oreoglanis siamensis from Klang river	.....
2. Oreoglanis siamensis from Klang Phat river	.....

B.

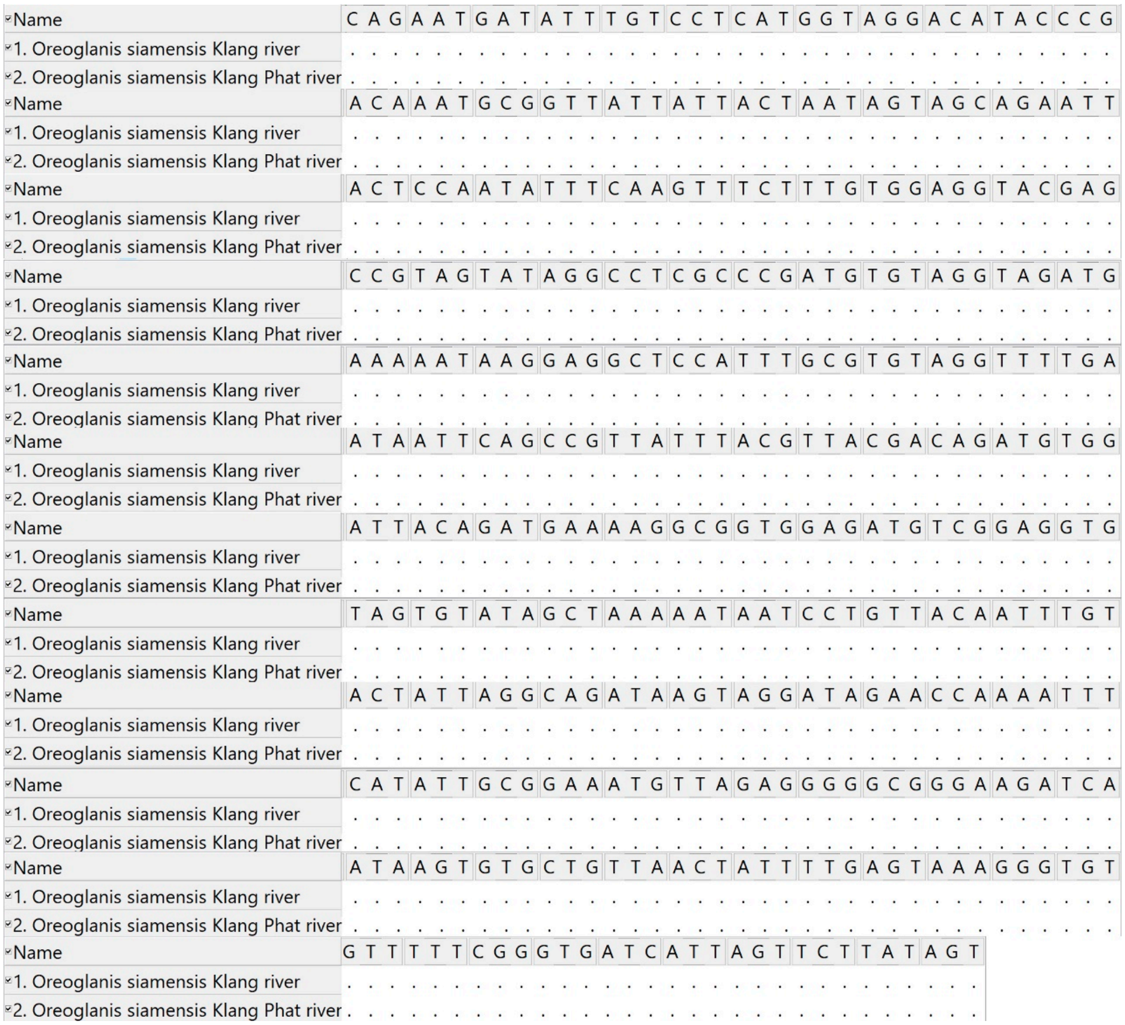
Name CAGAATGATATTTGTCCTCATGGTAGGACATACCCG
1. Oreoglanis siamensis Klang river
2. Oreoglanis siamensis Klang Phat river
Name ACAAATGCGGTTATTATTACTAATAGTAGCAGAATT
1. Oreoglanis siamensis Klang river
2. Oreoglanis siamensis Klang Phat river
Name ACTCCAATATTTCAAGTTCTTGTGGAGGTACGAG
1. Oreoglanis siamensis Klang river
2. Oreoglanis siamensis Klang Phat river
Name CCGTAGTATAGGCCTCGCCCGATGTGTAGGTAGATG
1. Oreoglanis siamensis Klang river
2. Oreoglanis siamensis Klang Phat river
Name AAAAATAAGGAGGCTCCATTTGCGTGTAGGTTTTGA
1. Oreoglanis siamensis Klang river
2. Oreoglanis siamensis Klang Phat river
Name ATAATTCAGCCGTTATTTACGTTACGACAGATGTGG
1. Oreoglanis siamensis Klang river
2. Oreoglanis siamensis Klang Phat river
Name ATTACAGATGAAAAGGCGGTGGAGATGTCGGAGGTG
1. Oreoglanis siamensis Klang river
2. Oreoglanis siamensis Klang Phat river
Name TAGTGTATAGCTAAAAATAATCCTGTTACAATTTGT
1. Oreoglanis siamensis Klang river
2. Oreoglanis siamensis Klang Phat river
Name ACTATTAGGCAGATAAGTAGGATAGAACCAAAATTT
1. Oreoglanis siamensis Klang river
2. Oreoglanis siamensis Klang Phat river
Name CATATTGCGGAAATGTTAGAGGGGCGGGAAGATCA
1. Oreoglanis siamensis Klang river
2. Oreoglanis siamensis Klang Phat river
Name ATAAGTGTGCTGTTAACTATTTTGAGTAAAGGGTGT
1. Oreoglanis siamensis Klang river
2. Oreoglanis siamensis Klang Phat river
Name GTTTTTCGGGTGATCATTAGTTCTTATAGT
1. Oreoglanis siamensis Klang river
2. Oreoglanis siamensis Klang Phat river

C.

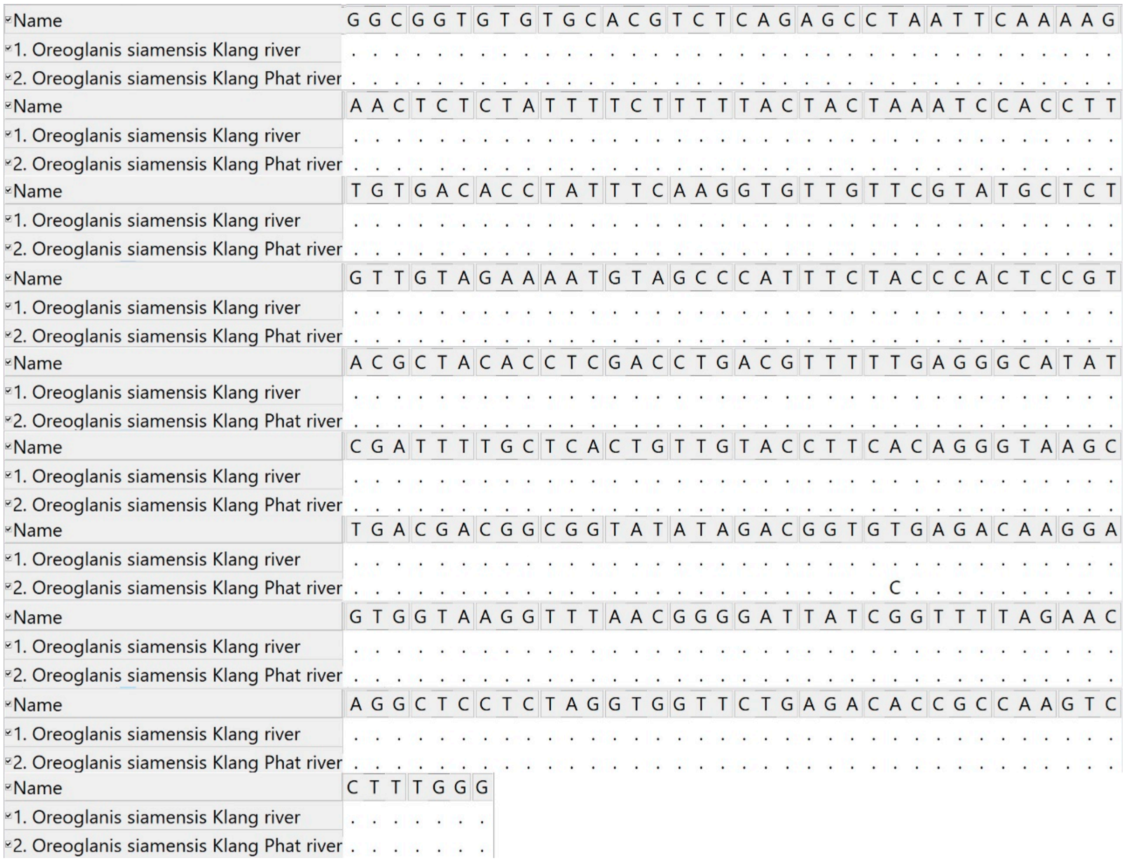
Name GGCGGTGTGTGCACGTCTCAGAGCCTAATTCAAAAG
1. Oreoglanis siamensis Klang river
2. Oreoglanis siamensis Klang Phat river
Name AACTCTCTATTTTCTTTTACTACTAAATCCACCTT
1. Oreoglanis siamensis Klang river
2. Oreoglanis siamensis Klang Phat river
Name TGTGACACCTATTTCAAGGTGTTGTTCGTATGCTCT
1. Oreoglanis siamensis Klang river
2. Oreoglanis siamensis Klang Phat river
Name GTTGTAGAAAATGTAGCCCATTTCTACCCACTCCGT
1. Oreoglanis siamensis Klang river
2. Oreoglanis siamensis Klang Phat river
Name ACGCTACACCTCGACCTGACGTTTTTGAGGGCATAT
1. Oreoglanis siamensis Klang river
2. Oreoglanis siamensis Klang Phat river
Name CGATTTTGCTCACTGTTGTACCTTCACAGGGTAAGC
1. Oreoglanis siamensis Klang river
2. Oreoglanis siamensis Klang Phat river
Name TGACGACGGCGGTATATAGACGGTGTGAGACAAGGA
1. Oreoglanis siamensis Klang river
2. Oreoglanis siamensis Klang Phat river C
Name GTGGTAAGGTTTAACGGGGATTATCGGTTTTAGAAC
1. Oreoglanis siamensis Klang river
2. Oreoglanis siamensis Klang Phat river
Name AGGCTCCTCTAGGTGGTTCTGAGACACCGCCAAGTC
1. Oreoglanis siamensis Klang river
2. Oreoglanis siamensis Klang Phat river
Name CTTTGGG
1. Oreoglanis siamensis Klang river
2. Oreoglanis siamensis Klang Phat river

D.

Name	Sequence
Name	CATACCTGCCCAGTGAAAATTTTTAAACGGCCGCGG
1. Oreoglanis siamensis Klang Phat river	....
2. Oreoglanis siamensis Klang river	....
Name	ATTCTAACCGTGCAAAGGTAGCGCAATCACTTGTCT
1. Oreoglanis siamensis Klang Phat river	....
2. Oreoglanis siamensis Klang river	....
Name	CTTAAATAGAGACCTGTATGAATGGTAAGACGAGGG
1. Oreoglanis siamensis Klang Phat river	....
2. Oreoglanis siamensis Klang river	....
Name	CTTAACTGTCTCCCTTTTCAAGTCAATGAAATTGAT
1. Oreoglanis siamensis Klang Phat river	....
2. Oreoglanis siamensis Klang river	....
Name	TGATCTGCCCGTGCAGAAGCGGACATAAAAATACAA
1. Oreoglanis siamensis Klang Phat river	....
2. Oreoglanis siamensis Klang river	....
Name	GACGAGAAGACCCTTTGGAACTTAAGACATCAGACC
1. Oreoglanis siamensis Klang Phat river	....
2. Oreoglanis siamensis Klang river	....
Name	CATATACATAATCCCAACATTCATTAACCTAATAGT
1. Oreoglanis siamensis Klang Phat river	....
2. Oreoglanis siamensis Klang river	T...................................
Name	AATTGGTCCCAGTCTTTGGTTGGGGCGACCGCGGAA
1. Oreoglanis siamensis Klang Phat river	....
2. Oreoglanis siamensis Klang river	....
Name	GAAAACAGAGCTACCGCGCAGATGGGGCAATCCCCT
1. Oreoglanis siamensis Klang Phat river	....
2. Oreoglanis siamensis Klang river	....
Name	AAAATCAAGAGAGACATCTCTAAATAACAGAACTTC
1. Oreoglanis siamensis Klang Phat river	....
2. Oreoglanis siamensis Klang river	....
Name	TGACCTTGAAGATCCGGCACTTACCGAACAACGAAC
1. Oreoglanis siamensis Klang Phat river	....
2. Oreoglanis siamensis Klang river	....
Name	CAAGCTACCCCAGGGATAACAGCGCAATCCCCTTTT
1. Oreoglanis siamensis Klang Phat river	....
2. Oreoglanis siamensis Klang river	....
Name	AGAGCCCATATCGACAAGGGGGTTTACGACCTCGAT
1. Oreoglanis siamensis Klang Phat river	....
2. Oreoglanis siamensis Klang river	....
Name	GTTGGATCAGGACATCCTAATGGTGCAGCCGCTATT
1. Oreoglanis siamensis Klang Phat river	....
2. Oreoglanis siamensis Klang river	....
Name	AAGGGTTCGTTTGTTCAACGA
1. Oreoglanis siamensis Klang Phat river	....
2. Oreoglanis siamensis Klang river	....
