# Supplementary material for: Detection of the Endangered Siamese Bat Catfish (Oreoglanis siamensis Smith, 1933) in Doi Inthanon National Park Using Environmental DNA
Source: Animals (Basel). 2023 Feb 3;13(3):538. doi: 10.3390/ani13030538 (PMC9913137; doi:10.3390/ani13030538)
Supplement: Supplementary file 1 [file animals-13-00538-s001.zip › animals-1939836-Supplementary Table S1.pdf]

Supplementary Table S1. GenBank accession numbers of species were used for primer design in this study.

Species	COI	Cytb	12S rRNA	16S rRNA
Amblyceps foratum	EU490875	EU490924		
Bagarius bagarius	MT812082	JQ413979		KT878056
Bagarius suchus	DQ846698			
Bagarius yarrelli	KM610424	AF416897	LC049878	AY445910
Bagrichthys majusculus		HQ257279	HQ257335	HQ257335
Bagrichthys obscurus		HQ257281	HQ257337	HQ257337
Barilius pulchellus	HM224207	MN541305	KU821706	KU884655
Cephalocassis borneensis		FJ626200	FJ626134	FJ626071
Channa striata	MW591031	MK258902	KU852423	MN380041
Crossocheilus reticulatus	MN342371	MH688196	MH688257	HM536783
Cyclocheilichthys repasson	MW147376	MH688190	MH688249	DQ464911
Danio albolineatus	MN342400	HM224262	LC193222	AF322663
Devario aequipinnatus	MT954943	HM224273	HM224273	KT835295
Devario annandalei	KY124375	HM224274		
Devario laoensis	MF172771	HM224278		
Discherodontus schroederi	JX066749	JX066767	LC069415	JX066733
Esomus metallicus	MW591064	EF151101	LC049901	FJ753467
Exostoma berdmorei	DQ846699			
Gyrinocheilus aymonieri	MN342601	MN541332	AY050541	AF357584
Hemibagrus filamentus	JQ289147	HQ257283	HQ257341	JQ248060
Hemibagrus spilopterus	MK049455	MN541353		
Hemibagrus wyckii	MG981082	HQ257287	HQ257345	HQ257345
Hemibagrus wyckioides	EU490862	EU490911	HQ257346	JQ248063
Mastacembelus tinwini	MW591093	KT944547		
Mystus albolineatus	KF824812	KF862963		
Mystus bocourti	EU490863	EU490912		JQ248058
Mystus multiradiatus	JX177677	HQ257295	HQ257353	JQ248055

Species	COI	Cytb	12S rRNA	16S rRNA
Mystus singaringan	MN992971	MN541357	MH688291	JQ248059
Neolissochilus stracheyi	MN096214	DQ464987	HQ699803	KT878231
Notopterus notopterus	JX983410	MZ381350	AF508062	LC388003
Opsarius koratensis	HM224205	HM224322	LC193403	
Opsarius pulchellus	HM224207	MN541305	MH688272	KU500876
Oreoglanis immaculatus	JQ859840			
Oreoglanis macropterus		DQ192479		
Oreoglanis siamensis	MZ753673	MZ773400	MZ766142	MZ766143
Pareuchiloglanis anteanalis	DQ508085	AY191610		AY445903
Pareuchiloglanis feae		JN986971		
Pareuchiloglanis hupingshanensis		KU356571		
Pareuchiloglanis myzostoma	MH853829			
Pareuchiloglanis sinensis	MF122630	DQ192484		AY445904
Pethia stoliczkana	MN342718	MT483275	LC193389	KP712685
Pseudomystus siamensis				
Raiamas guttatus	MK116352	HM483378		
Rasbora paviana	MW147436	MN541324	MH688286	U21554
Scaphiodonichthys acanthopterus	KJ994655	KJ994707		KP712612
Schistura poculi	MG238142	MG238239		
Schistura pridii		KP738603		
Systomus rubripinnis	MK448198			
Tor tambroides	KU692919	HM536824	HQ699796	AY973162
