# Supplementary material for: Detection of the Endangered Siamese Bat Catfish (Oreoglanis siamensis Smith, 1933) in Doi Inthanon National Park Using Environmental DNA
Source: Animals (Basel). 2023 Feb 3;13(3):538. doi: 10.3390/ani13030538 (PMC9913137; doi:10.3390/ani13030538)
Supplement: Supplementary file 1 [file animals-13-00538-s001.zip › animals-1939836-Supplementary Table S2.pdf]

Supplementary Table 2. Geographic coordinates of the sampling sites.

Sampling site	River	Geographic coordinates
KP1		18.560556, 98.544889
KP2	Klang Phat	18.560139, 98.545583
KP3		18.544389, 98.564500
KP4		18.54145, 98.5728900
K1		18.546560, 98.513250
K2		18.548270, 98.517550
K3		18.544900, 98.517620
K4	Klang	18.542000, 98.523180
K5		18.538390, 98.525160
K6		18.536310, 98.523100
K7		18.525210, 98.522250
K8		18.541889, 98.550111
K9		18.541690, 98.599690
K10		18.507180, 98.661670
K11		18.495270, 98.667570
