# Supplementary material for: Detection of the Endangered Siamese Bat Catfish (Oreoglanis siamensis Smith, 1933) in Doi Inthanon National Park Using Environmental DNA
Source: Animals (Basel). 2023 Feb 3;13(3):538. doi: 10.3390/ani13030538 (PMC9913137; doi:10.3390/ani13030538)
Supplement: Supplementary file 1 [file animals-13-00538-s001.zip › animals-1939836-Supplementary Table S3.pdf]

**Supplemental Table S3.** Overview of sequence alignments (160 bp) between positive qPCR amplicons from eDNA assays and *O. siamensis* gDNA sequence. One eDNA amplicon was randomly selected for sequencing from each sampling site for each sampling year (gray highlighted).

| Site | eDNA detection (15 Replicates) | | | | | | | | | | | | | | | Positive qPCR replicates | Sequence similarity between the amplicon and the target species gDNA (%) |
|---|---|---|---|---|---|---|---|---|---|---|---|---|---|---|---|---|---|
| | 2019 | | | | | 2020 | | | | | 2021 | | | | | | |
| KP1 | ✓ | ✓ | ✓ | ✓ | x | ✓ | ✓ | ✓ | ✓ | ✓ | ✓ | ✓ | ✓ | ✓ | ✓ | 14/15 | 100 |
| KP2 | ✓ | ✓ | ✓ | ✓ | ✓ | ✓ | x | ✓ | ✓ | ✓ | ✓ | ✓ | ✓ | ✓ | ✓ | 14/15 | 100 |
| KP3 | ✓ | ✓ | ✓ | ✓ | x | ✓ | ✓ | ✓ | ✓ | ✓ | ✓ | ✓ | ✓ | ✓ | x | 13/15 | 100 |
| KP4 | ✓ | ✓ | ✓ | ✓ | ✓ | ✓ | ✓ | ✓ | ✓ | ✓ | ✓ | ✓ | ✓ | ✓ | ✓ | 15/15 | 100 |
| K1 | ✓ | x | ✓ | x | ✓ | x | ✓ | x | ✓ | ✓ | ✓ | x | x | ✓ | ✓ | 9/15 | 100 |
| K2 | x | ✓ | ✓ | ✓ | ✓ | ✓ | x | x | ✓ | ✓ | ✓ | ✓ | ✓ | x | ✓ | 11/15 | 100 |
| K4 | ✓ | ✓ | x | x | ✓ | x | ✓ | ✓ | x | ✓ | x | ✓ | x | ✓ | ✓ | 9/15 | 100 |
| K5 | ✓ | ✓ | ✓ | ✓ | x | ✓ | ✓ | x | ✓ | ✓ | ✓ | ✓ | ✓ | ✓ | ✓ | 13/15 | 100 |
| K7 | x | ✓ | x | ✓ | ✓ | ✓ | ✓ | ✓ | ✓ | x | ✓ | ✓ | ✓ | ✓ | x | 11/15 | 100 |
| K8 | ✓ | x | x | ✓ | ✓ | ✓ | ✓ | ✓ | ✓ | ✓ | ✓ | ✓ | ✓ | x | x | 11/15 | 100 |
| K9 | ✓ | ✓ | ✓ | x | ✓ | ✓ | x | ✓ | x | ✓ | ✓ | x | ✓ | ✓ | ✓ | 11/15 | 100 |
| K10 | ✓ | ✓ | ✓ | ✓ | ✓ | ✓ | ✓ | ✓ | ✓ | ✓ | ✓ | ✓ | ✓ | x | ✓ | 14/15 | 100 |
